# The functions and targets of miR‐212 as a potential biomarker of cancer diagnosis and therapy

**DOI:** 10.1111/jcmm.14966

**Published:** 2020-01-13

**Authors:** Wenjun Chen, Jing Song, Hongjun Bian, Xia Yang, Xiaoyu Xie, Qiang Zhu, Chengyong Qin, Jianni Qi

**Affiliations:** ^1^ Departments of Gastroenterology Shandong Provincial Hospital Affiliated to Shandong University Jinan China; ^2^ Shandong Provincial Engineering and Technological Research Center for Liver Diseases Prevention and Control Jinan China; ^3^ Departments of Gastroenterology The Affiliated Weihai Second Municipal Hospital of Qingdao University Qingdao China; ^4^ Departments of Emergency Medicine Shandong Provincial Hospital Affiliated to Shandong University Jinan China; ^5^ Central Laboratory Shandong Provincial Hospital Affiliated to Shandong University Shandong Provincial Hospital Affiliated to Shandong First Medical University Jinan China

**Keywords:** Hedgehog, Hippo/YAP, miR‐212, oncogenesis, Wnt

## Abstract

Cancer is a major health problem worldwide. An increasing number of researchers are studying the diagnosis, therapy and mechanisms underlying the development and progression of cancer. The study of noncoding RNA has attracted a lot of attention in recent years. It was found that frequent alterations of miRNA expression not only have various functions in cancer but also that miRNAs can act as clinical markers of diagnosis, stage and progression of cancer. MiR‐212 is an important example of miRNAs involved in cancer. According to recent studies, miR‐212 may serve as an oncogene or tumour suppressor by influencing different targets or pathways during the oncogenesis and the development and metastasis of cancer. Its deregulation may serve as a marker for the diagnosis or prognosis of cancer. In addition, it was recently reported that miR‐212 was related to the sensitivity or resistance of cancer cells to chemotherapy or radiotherapy. Here, we summarize the current understanding of miR‐212 functions in cancer by describing the relevant signalling pathways and targets. The role of miR‐212 as a biomarker and its therapeutic potential in cancer is also described. The aim of this review was to identify new methods for the diagnosis and treatment of human cancers.

## 1 INTRODUCTION

For many years, cancer has been the main cause of death worldwide.^1^ Recently, a large number of researchers have devoted themselves to the identification of targets or pathways involved in cancer and the development of targeted therapies that may efficiently kill cancer cells. Abnormal expression of miRNAs can be seen in different types of human cancers and correlates with proliferation, differentiation, invasion and other biological aspects of cancer.^2^

miRNAs, about 18‐25 nucleotides in length, are non‐coding RNA molecules that can regulate the expression of genes involved in various biological processes, including proliferation, apoptosis, differentiation and survival of cellular processes, at the post‐transcriptionally level.^3^, ^4^ miRNAs can participate in both physiological and pathological processes, such as tumours development, via interaction with the 3′ untranslated regions (UTRs) of their target mRNAs.^5^, ^6^ According to the role they play in cancers, miRNAs are classified as tumour promoters or tumour suppressors.^7^ For instance, miR‐802, as an oncogenic miRNA, promotes the proliferation of osteosarcoma (OS) cells via the suppression of p‐27, which is a kind of cell‐cycle inhibitor,^8^ while miRNA‐223 acts as a tumour suppressor in non–small‐cell lung cancer (NSCLC) by interacting with the insulin‐like growth factor‐1 receptor.^9^ It was also recently demonstrated that miRNAs may play a role in drug resistance.^10^

miR‐212, found on chromosome 17q13.3, shares highly conserved sequences with miR‐132 among vertebrates. miR‐132 and miR‐212 play an essential role in different aspects of neural development, maturation, morphogenesis and function. Therefore, the abnormal expression of miR‐132 and miR‐212 may cause a series of neurodegenerative diseases, such as Alzheimer's disease, epilepsy, tauopathies and schizophrenia. Thus, miR‐132/212 is sometimes called ‘neurimmiR’.^11^ Although the initial research on miR‐212 emerged from studies performed in the neuronal context as well as studies on inflammation and other biological (dys)functions, studies on tumorigenesis and cell transformation have become the most popular in recent miR‐212 research.^11^ More and more studies are reporting the abnormal expression of miR‐212 in various cancers and that miR‐212 can act as a marker to diagnose or predict the outcome of cancer. In previous studies, miR‐212‐3p/5p was reported to be suppressed in hepatocellular carcinoma (HCC),^12^ ovarian cancer 13 and gastric cancer (GC) 14 and showed antitumour effect. Two other studies indicated that miR‐212 functions as an oncogene, with a higher expression in pancreatic cancer.^15^, ^16^ The different functions performed by miR‐212 in cancer depend on the tumour types, targets or pathways involved.^17^ In addition, evidence has emerged that the decreased expression of miR‐212‐3p may be due to DNA hypermethylation, which weakens the effect of miR‐212‐3p and regulates the biological characteristics of tumour cells.^18^ However, another study found that it was histone modifications, not DNA hypermethylation that led to the decreased expression of miR‐212‐3p in lung cancer.^19^ Recently, growing evidence has indicated that miR‐212 affects the response to radiotherapy or chemotherapy. This review discusses the pathways or targets of miR‐212 involved in cancer, summarizes its function as a marker for the diagnosis or prediction of the outcome of cancer and, finally, analyses the therapeutic potential of miR‐212 for malignant tumours.

## 2 THE ROLE OF MIR‐212 IN CANCER

### 2.1 miR‐212 and cell apoptosis

Apoptosis, a process characterized by programmed cell death, occurs in both the physiological state, such as for maintaining cell populations in tissues, as well as pathological states, such as during injured cells.^20^, ^21^ Both intrinsic and extrinsic pathways may lead to apoptosis. When stimulated by specific growth factors or cytokines, apoptosis usually occurs through the extrinsic pathway. To our knowledge, not only precancerous cells, but also tumour cells can be induced to undergo apoptosis by p53.^22^ Recent research showed that miR‐212 is associated with cancer cells apoptosis through various targets.

A study performed by Zhou et al^23^ demonstrated that overexpression of miR‐212 inhibited prostate cancer (PCa) cell proliferation and induced PCa cell apoptosis via targeting EN‐2, whereas the restoration of EN‐2 led to the opposite effect. Lu et al^24^ verified that miR‐212‐3p inhibited the apoptosis induced by cisplatin through interaction with AChE‐S in NSCLC cells. A study performed by Hai‐hao Wang revealed that overexpression of miR‐212‐5p significantly suppressed the expression of the CCND3 protein by targeting the CCND3 gene and that upregulation of CCND3 positively correlated with cell growth in miR‐212‐5p overexpressing adult T‐cell leukaemia/lymphoma cells, which was accompanied by restoration of cell cycle progression and reduction of apoptotic death.^25^ It was also reported that CCND3, encoding protein cyclin D3, played an important role in controlling the advancement through the G0/G1 phase of the cell cycle.^26^ XIAP, a key molecule of the cell apoptosis pathway, could prohibit cell apoptosis by inhibiting caspase‐3, caspase‐7 and caspase‐9.^27^, ^28^, ^29^ In renal cell carcinoma (RCC), it was found that miR‐212‐5p inhibited the progression of RCC cells via interaction with XIAP.^30^ The remarkable mechanism of competing endogenous RNA (ceRNA) has recently attracted a lot of attention. One study found that miR‐212‐5p may induce degradation or inhibit the expression of NDUFA4. Moreover, LncMIF‐AS1 was found to promote the expression of NDUFA4 in GC cells by binding miR‐212‐5p, and the recovered NDUFA4 strengthened the proliferative ability and prevented apoptosis of GC cells in vitro.^31^

### 2.2 miR‐212 and the cell cycle

A number of molecular pathways and checkpoints regulate the cell cycle in most adult mammalian cells. Many miRNAs take part in these pathways. For example, some miRNAs are involved in the anti‐proliferation of cells by inhibiting mitogenic pathways responsive to the activation of cyclin‐CDK complexes. In contrast, other miRNAs may facilitate tumour cell proliferation by interacting with CDK inhibitors, such as p53 and ATM/ATR.^32^ SIRT1, which was shown to modulate both physiological and pathological processes in cells via acting on cell cycle proteins, including Rb, is now verified to be targeted by miR‐212 in some cancers.^33^, ^34^ miR‐212‐3p was shown by Ramalinga et al^35^ to prevent autophagy and angiogenesis while inducing cellular senescence by targeting SIRT1 in PCa. Likewise, it was demonstrated that miR‐212‐3p can suppress thyroid cancer cell growth through SIRT1.^36^ One study conducted by Bo Hu demonstrated that miR‐212‐3p has an antitumour role in PCa by means of targeting MAPK1^37^. MAPK1, which belongs to the MAPK family, is closely associated with PCa, especially in the aspects of cell proliferation, cell cycle and apoptosis.^38^ Ji‐ping Zeng demonstrated that miR‐212‐3p suppressed GC growth by modulating P21^CIP1^ and P27^kip1^.^39^ It was verified that P21^CIP1^ and P27^kip1^ are associated with cell cycle arrest, and they showed a tendency for lower expression in GC.^40^ Jong‐Kook Park found that upregulated miR‐132‐3p/‐212‐3p in pancreatic ductal adenocarcinoma (PDAC) increased cell proliferation via targeting the tumour suppressor Rb1.^15^ pRb, a protein produced by the gene Rb1, participates in the regulation of the cell cycle, specifically the transition of G1/S and G2/M.^41^ In HCC, it is suggested that downregulated hsa‐miR‐212‐3p expression can lead to overexpression of RBP2 and promote cell proliferation. The authors of the study that revealed this concluded that there was a link between the hsa‐miR‐212‐3p/RBP2/CDKI pathways and progression of HCC.^42^ Likewise, for lung cancer cells, miR‐212/132 can inhibit the development of tumour cells and affect the cell cycle by targeting p21 and cyclin D1.^43^ Thus, functional studies of miR‐212 can help us better understand the role of miR‐212 in cell cycle regulation.

### 2.3 miR‐212 and immune responses

miRNAs participate in both innate and adaptive immunity by interacting with various immune cells, including monocytes, macrophages, NK cells and T helper cells, in differentiation, activation and other functions. The relationship between miRNAs and immunity has important effects on cancer progression.^44^

An earlier study on the biological functions of miRNA‐212 found it to interact with the neurons as well as inflammation.^11^ Studies performed recently demonstrated that miRNA‐212‐3p can interact with immune cells and inflammatory cytokines in the occurrence or development of cancer. It was shown that downregulated miRNA‐212‐3p regulates the development of PCa through the secretion of inflammatory cytokines via the NF‐κB pathway.^45^ Another study showed that the regulatory effect of miR‐212‐3p in CD80 expression can be disrupted by the SNP rs1599795 in the 3′‐UTR of CD80, which may induce GC tumorigenesis.^46^ Ding et al^47^ suggested that IFN‐γ could be used as an immunological method to treat pancreatic cancer as it inhibits miR‐212‐3p expression and the subsequent upregulation of RFXAP and MHC class II. It was also found that miR‐212‐3p may induce immunologic tolerance of pancreatic tumour cells by affecting dendritic cell functions.^48^ The findings of these studies indicate that targeting miR‐212 will become a promising cancer immunotherapy approach.

### 2.4 miR‐212 as a diagnostic or prognostic biomarker

Multiple data indicate that miRNAs participate in human carcinogenesis. It was shown that some miRNAs were significantly upregulated or downregulated in comparison with paired normal tissues in various cancers, emphasizing the tremendous potential of miRNAs as diagnostic and therapeutic markers in cancer. Additionally, some miRNAs were found to be linked with the outcome of certain cancers. Importantly, these miRNAs can exist either in cells or in cell‐free body fluids such as urine and saliva. This stability of miRNAs in body fluids allows them to be easily tested as biomarkers of disease.^49^

Emerging researches have reported that the aberrant expression of miR‐212 can be seen in different cancers and can be used for the diagnosis or prediction of outcome of cancer. One study on 386 patients revealed that a miRNA signature, including hsa‐miR‐212, may help identify early‐stage breast cancer.^50^ After examining 45 PDAC and 20 adjacent normal pancreatic tissue specimens, miR‐212‐3p was found to correlate with tumour growth and disease stage in PDAC, and high levels of miR‐212‐3p were associated with poor outcome.^51^ In oesophageal cancer (EC), one study involving 46 patients revealed that, with other factors, such as treatment or staging of comparable disease, high miR‐212 expression was accompanied with poor outcome.^52^ Contrarily, in acute myeloid leukaemia (AML), according to a study involving 576 patients, the high expression of miR‐212 was associated with a better outcome of patients and its role in predicting cancer was found to not be affected by other factors.^53^ One study, including 15 cases associated with lymph node (LN) metastasis and another 15 cases without LN metastasis, found that there was a link between downregulated hsa‐miR‐212 expression and LN metastasis in GC and suggested that the level of hsa‐miR‐212 may serve as a clinic marker for GC.^54^ The effect of miR‐212 on lung cancer is controversial. One study involving 418 lung adenocarcinoma (LUAD) patients found that high miR‐212 levels predicted a poor recurrence‐free survival (RFS),^55^ whereas another study, including 34 adenocarcinoma and squamous cell carcinoma tissue samples, indicated that low expression of miR‐212 in patients represented the severity of the disease.^19^ This discrepancy may be caused by the different histological types of lung cancer involved in these studies. More researches enrolling a larger number of patients with a unified disease type are required to elucidate the controversial role of miR‐212 in lung cancers. In breast cancer, after comparing the expression of miR‐212‐5p in 125 triple‐negative breast cancer (TNBC) patients, it was revealed that the low expression of miR‐212‐5p predicted an unfavourable outcome.^56^ miRNAs can be tested not only in cells but also in cell‐free body fluids, such as saliva and urine. In a study involving 173 HCC patients, serum miR‐132/212 was shown to assist in diagnosis and prediction of the progression as well as outcome of HCC.^57^ Chao‐hui Gu studied samples from 60 patients with RCC and found that a poor outcome was often indicated in RCC patients with down‐regulated miR‐212‐5p.^30^ In chronic lymphocytic leukaemia (CLL), Tavolaro et al^58^ evaluated the basal expression of miR‐212 in 20 CLL cases and found that the downregulation of miR‐132 and miR‐212 was associated with progressive disease and a poor prognosis of CLL. A summary of the diagnostic and prognostic functions of miR‐212 is shown in Table 1.

**Table 1:** Overview of down‐and upregulation of miR‐212 with possible diagnostic and prognostic role in different cancers

miR‐212	Level	Cancer types	Diagnosis or prognosis	Ref.
miR‐212	Up	Breast cancer	Poor prognosis	85
miR‐212‐3p	Up	PDAC	Poor prognosis	86
miR‐212	Up	Esophageal cancer	Poor prognosis	87
miR‐212	Up	AML	Better survival	88
miR‐212	Up	Gastric cancer	Lymph node metastasis	89
miR‐212	Up	LUAD	Poor prognosis	90
miR‐212	Down	NSCLC	Poor prognosis	91
miR‐212‐5p	Down	TNBC	Poor prognosis	23
miR‐212	Down	Hepatocellular carcinoma	Improved diagnosis, Poor prognosis	92
miR‐212‐5p	Down	Renal cell carcinoma	Poor prognosis	52
miR‐212	Down	CLL	Poor prognosis	93

Abbreviations: AML, acute myeloid leukaemia; CLL, chronic lymphocytic leukaemia; LUAD, lung adenocarcinoma; NSCLC, non–small‐cell lung carcinoma; PDAC, pancreatic ductal adenocarcinoma; TNBC, triple‐negative breast cancer.

### 2.5 miR‐212 and cancer treatments

Both chemotherapy and radiotherapy are important treatments for cancer and growing evidence shows that the response to chemotherapy or radiotherapy is affected by miRNAs. Recently, miR‐212 was found or proposed to participate in the regulation of cancer sensitivity to chemotherapy or radiotherapy. Xie et al^59^ found that miR‐132/‐212‐3p may induce drug resistance of in breast cancer via the miR‐132/‐212‐3p/PTEN/AKT/NF‐κB/BCRP pathway. Further, they suggested that it could improve the treatment effects of doxorubicin, and they identified novel therapeutic targets for breast cancer by modulating miR‐132/‐212‐3p. In contrast, another study found that miR‐212‐3p increases the sensitivity of NSCLC cells to TRAIL because there is a negative relationship between miR‐212‐3p and PED/PEA‐15.^60^ Turrini et al^61^ showed that imatinib downregulated miR‐212‐3p expression, causing the restoration of ABCG2. They showed a regulatory relationship between imatinib treatment, miRNA and ABCG2 in vitro for the first time. Further research is necessary to illuminate this link in vivo. According to a study by Hiromitsu Hatakeyama, down‐regulated miR‐212‐3p may augment the expression of HB‐EGF, which is a possible mechanism of drug resistance of head and neck squamous cell carcinoma cells to cetuximab.^62^ With regard to radiosensitivity, it was reported that the BRCA1 gene is regulated by miR‐212‐3p and affected the sensitivity of glioma cells to radiotherapy. More studies should, thus, focus on the role of miR­212‐3p and BRCA1 in radioresistance.^63^ With the progress of research on miR‐212, a miR‐212‐based treatment will be available for cancer. For example, the expression of miR‐212 can be replaced or reduced to alter the response of cancer cells to chemotherapy or radiotherapy. Excitingly, further experiments utilizing miR‐212 to treat cancer are on the way.

## 3 THE PATHWAYS AND TARGETS OF MIR‐212 IN CANCER

### 3.1 The Wnt signalling pathway

**Figure 1:**
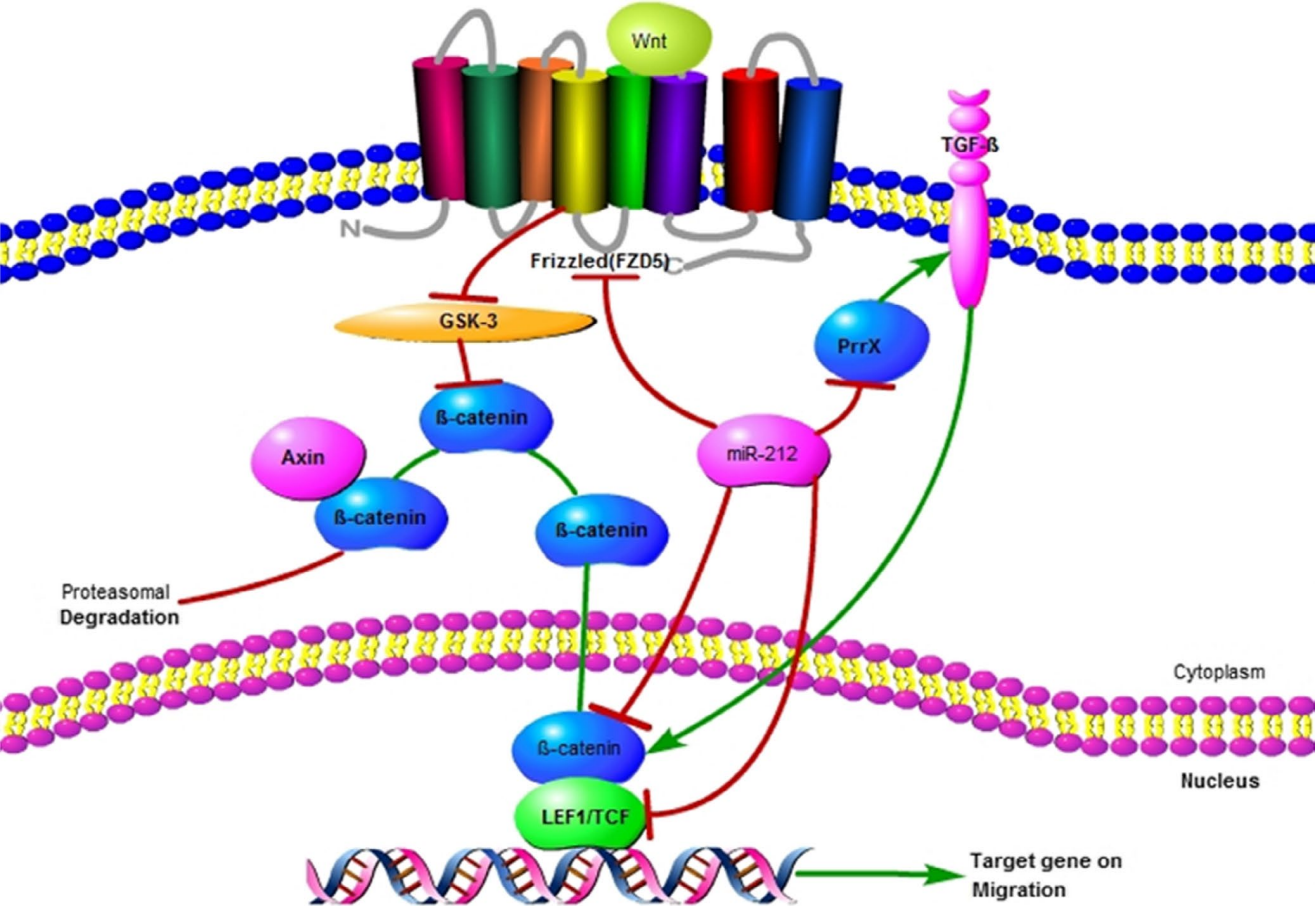
miR212 and Wnt/β‐catenin signaling

The dysregulation of the Wnt/β­catenin pathway is believed to be central to the pathogenesis of cancer, chronic inflammation and degenerative diseases. The phosphorylation, degradation and regulation of β­catenin by Wnt are considered to form the core of this pathway. Without Wnt, Wnt target genes are significantly inhibited because of the persistent degradation of the β­catenin protein by the Axin complex.^64^, ^65^, ^66^ It was reported that the dysregulation of miR‐212 participated in different cancers through the Wnt/β­catenin pathway via specific targets (Figure 1).

Zhi‐dong Lv and colleagues showed that miR‐212‐5p may target Prrx2, which plays an important role in the proliferation, invasion and migration of breast cancer cells via the Wnt/β‐catenin signalling pathway.^56^ Recently, miR‐212‐3p was found to regulate Wnt signalling pathways by acting on LEF‐1, c‐Myc and β‐catenin in HCC, which is why miR‐212‐3p can inhibit the tumorigenesis and growth of HCC cells.^67^ The inhibition of the Wnt/β‐catenin signalling pathway by miR‐212‐5p was also found in AML, and FZD5 was reported as the target gene of miR‐212‐5p.^68^ Zhou C showed that miR‐212‐3p suppressed cervical cancer through the downregulation of its downstream target gene TCF7L2,^69^ while TCF7L2 is thought to be the pivotal element in the Wnt signalling pathway.^70^, ^71^

### 3.2 The Hedgehog signalling pathway

The Hedgehog (Hh) pathway has as a role in embryonic development, tissue patterning and wound healing.^72^ Aberrant functioning of this pathway is connected with the development of cancer in several organs.^73^, ^74^ The Hh pathway is often triggered by ligands. SHH, one of the identified ligands of the Hh pathway, is the most studied.^75^ Without SHH, PTCH1 can hinder the expression of smoothened (SMO), a target protein of the Hh pathway. As a result of the interaction between SHH and PTCH1, SMO and the subsequent activation of GLi transcription factors can be reduced, and the functions of Gli1, Gli2 and Gli3 are affected in the end.^75^ Thus, PTCH1 is called the “gatekeeper” of the Hh pathway.

**Figure 2:**
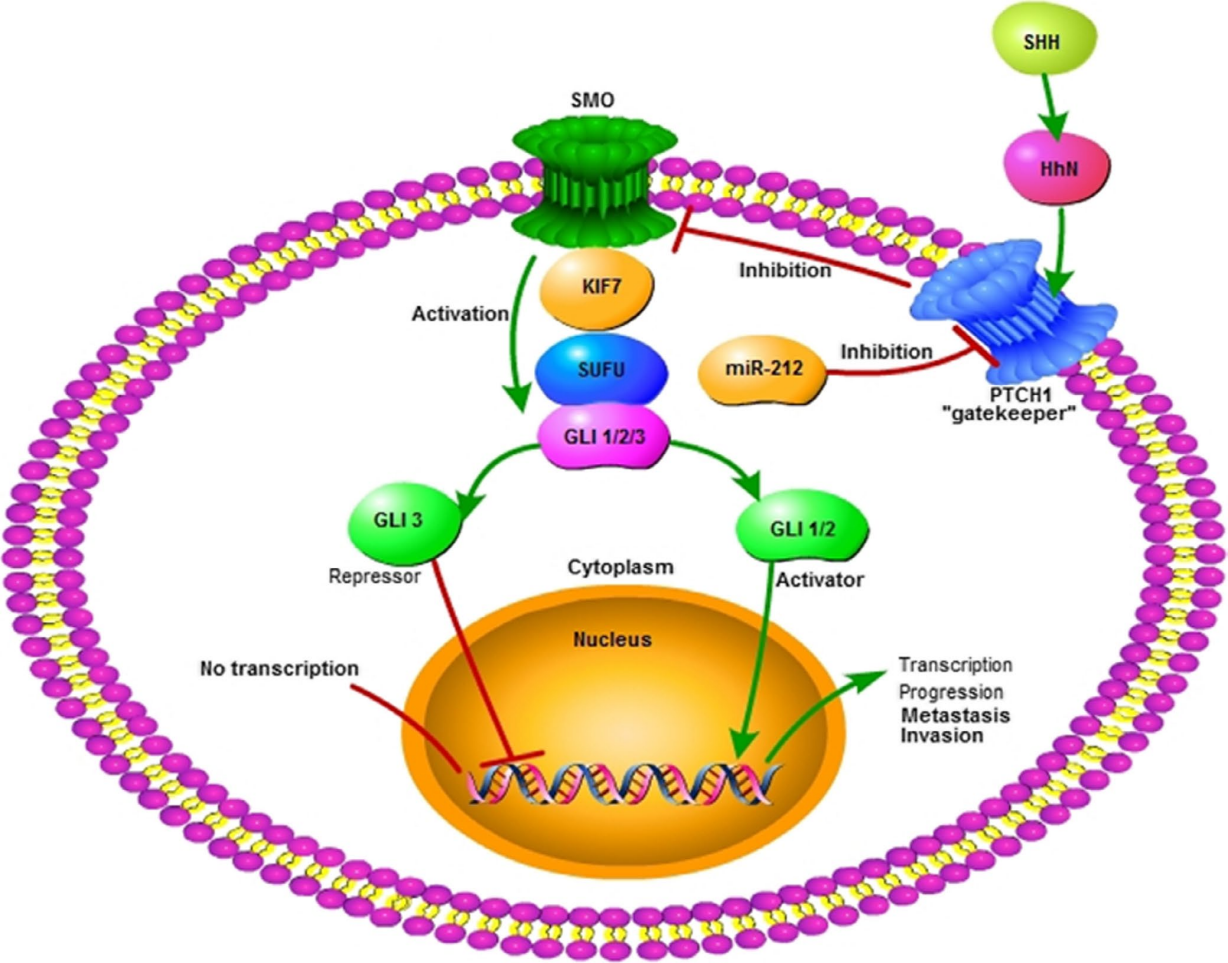
miR212 and Hedgehog pathway

In some kinds of cancer, miR‐212 may play a role as an oncogene targeting PTCH1 (Figure 2). miR‐212‐3p was shown by Chen‐chao Ma and colleagues to promote PDAC progression and metastasis via modulation of the Hh signalling pathway receptor PTCH1.^16^ In the case of NSCLC, Yuan Li found miR‐212‐3p may act as an oncogene to promote cell proliferation and other aggressive behaviour of tumour cells by acting on PTCH1.^76^

### 3.3 The Hippo/YAP signalling pathway

The Hippo/YAP signalling pathway was found to be associated with liver size.^77^, ^78^ YAP and TAZ, the main effectors of the Hippo signalling pathway, are controlled by a series of kinase cascades. The main proliferative and oncogenic function performed by YAP and TAZ is associated with the members of the TEAD/TEF transcription factor family (TEAD1‐4).^79^ For example, it was reported that YAP has the potential to induce liver tumorigenesis.^80^ FOXA1 has been previously reported to promote YAP transcription by Wen‐jun Yu.^81^ Recently, the relationship between miR‐212 and FOXA1 was reported in various cancers.

**Figure 3:**
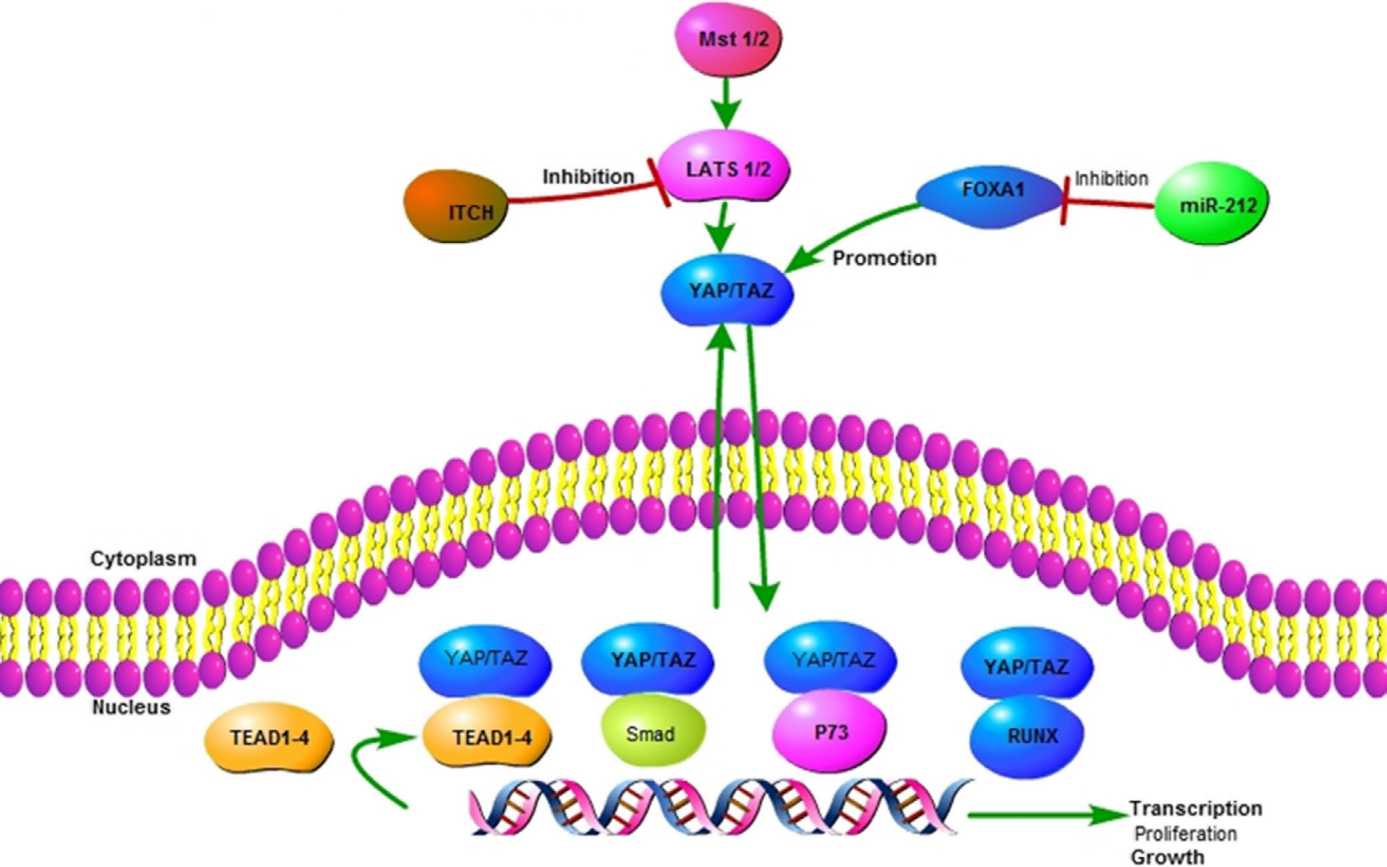
miR212 and Hippo/YAP signaling in HCC

Dou et al^12^ reported that miR‐212‐3p repressed the expression of YAP via FOXA1 in HCC, emphasizing the significance of the miR‐212‐3p/FOXA1/Hippo/YAP pathway in HCC, (Figure 3). In accordance with this finding, Hua‐hua Tu demonstrated that miR‐212‐3p can inhibit HCC cell proliferation by targeting FOXA1.^17^ In OS, Chu‐hai Xie reported that TUG1 can sponge miR‐212‐3p to upregulate FOXA1, with the subsequent effect of regulating cell proliferation and apoptosis in tumour cells.^82^ Liu et al^83^ suggested that the anti‐migration effect of miR‐212‐3p in OS cells was implemented via targeting FOXA1. In the latter two studies, although FOXA1 was the target of miR‐212‐3p, whether they are all involved in the same FOXA1/Hippo/YAP pathway requires further study.

### 3.4 The other targets of miR‐212

miRNAs participate in various physiological and pathological processes by interacting with their target mRNAs. Apart from the pathways described above, miR‐212 acts on a series of targets. The interaction between miR‐212 and its targets may result in different functions depending on the target or tumour types. The targets and the effects of miR‐212 are clearly shown in Table 2.

**Table 2:** Targets of miR‐212 known to be involved in cancers

No.	miR‐212	Target genes	Cancer types	Related functions	Ref.
1	3p	USP9X	NSCLC	Migration, invasion	70
2	3p	USP9X	PDAC	EMT, apoptosis, autophagy	71
3	3p	SOX4	Breast cancer	Metastasis	72
4	3p	SOX4	Prostate cancer	Invasion, metastasis	73
5	5p	SOX4	NSCLC	Migration, invasion	74
6	5p	SOX4	Osteosarcoma	Proliferation, Invasion	75
7	3p	SOX4	NPC	Migration, invasion	76
8	3p	SOX4	Ovarian cancer	EMT	77
9	3p	MnSOD	Colorectal cancer	Metastasis	78
10	3p	ZO‐1	Colorectal cancer	Metastasis	79
11	3p	SMAD2	Cervical cancer	Cell growth, migration, invasion	80
12	3p	PXN	Gastric cancer	Metastasis and invasion	18
13	3p	MeCP2	Gastric carcinoma	Carcinogenesis	81
14	3p	Lin28B	AIPC	Carcinogenesis	12
15	3p	SGK3	GBM	Proliferation	82
16	3p	HBEGF	Ovarian cancer	Proliferation, migration, invasion	13

Abbreviations: AIPC, androgen‐independent prostate cancer; GBM, glioblastoma multiforme; NPC, nasopharyngeal carcinoma; NSCLC, non–small‐cell lung cancer; PDAC, pancreatic ductal adenocarcinoma.

According to the Table 2, USP9X and SOX4 are the common targets of miR‐212‐3p or ‐5p in different tumour types,^84^, ^85^, ^86^, ^87^, ^88^, ^89^, ^90^, ^91^ whereas miR‐212 may target different genes having different functions in colorectal cancer and NSCLC.^84^, ^88^, ^92^, ^93^ Moreover, in cervical cancer, miR‐212‐3p was reported to play a role of tumour suppressor via targeting SMAD2.^94^ Meanwhile, in gastric cancer, miR‐212‐3p was indicated to take part in gastric carcinogenesis through targeting MeCP2.^14^ And in androgen‐independent prostate cancer (AIPC), miR‐212‐3p and Lin28B were reported to form a potential regulatory loop to modulate c‐Myc and prostate carcinogenesis.^95^ Yet, in glioblastoma multiforme (GBM), miR‐212‐3p was revealed to suppress the proliferation of GBM cells via targeting SGK3.^96^ In addition, miR‐212‐3p/miR‐132‐3p and SOX4 can interact with each other, creating a feedback loop in ovarian cancer cells. Specifically, the SOX4/EZH2 complex can silence miR‐212‐3p/132‐3p expression, while miR‐212‐3p and miR‐132‐3p can inhibit the expression of SOX4 and modulate EMT of ovarian cancer cells.^91^ According to computer prediction, each miRNA can regulate about 200 mRNAs. Meanwhile, one protein‐encoding gene also can also be modulated by multiple miRNAs, indicating that miRNAs are important regulators of mRNAs.^97^ Thus, it is essential to further explore the regulatory network of miRNA‐212 in different human cancers.

## 4 CONCLUSION AND PERSPECTIVES

In general, a plenty of evidences show that miR‐212 can interact with different targets and participate in multiple pathways, as well as immune responses, in cancer. Dysregulated miR‐212 may function as a promoter or suppressor in different aspects of tumorigenesis including cell proliferation, invasion, metastasis and apoptosis. Both DNA hypermethylation and histone modification may lead to the dysregulation of miR‐212 in different cancers. MiR‐212, downregulation or upregulation, may act as a biomarker for the diagnosis or prognosis of cancer. Especially, since miR‐212 can also be detected in serum and plasma, which are much more readily obtainable than tissues, it attracts increased clinical attention as a biomarker. The controversial findings regarding miR‐212 in some cancers require further study. miR‐212 participates in chemoresistance and radioresistance, significantly affecting cancer treatment. Identifying vital miR‐212 targets and developing safe, effective methods to address miR‐212 involvement in the resistance to chemotherapy or radiotherapy will become an important focus in the field of cancer therapy.

## CONFLICTS OF INTEREST

The authors declare that they have no conflicts of interest.

## AUTHOR CONTRIBUTIONS

Conception and design: Jianni Qi and Hongjun Bian. Collection and assembly of data: Wenjun Chen, Jing Song, Xia Yang, Xiaoyu Xie. Data analysis and interpretation: Wenjun Chen and Hongjun Bian. Manuscript writing: Jianni Qi and Wenjun Chen. Administrative support: Qiang Zhu and Chengyong Qin. Final approval of manuscript: All authors.
